# Exsolved catalyst particles as a plaything of atmosphere and electrochemistry

**DOI:** 10.1039/d2ey00036a

**Published:** 2023-03-20

**Authors:** Harald Summerer, Andreas Nenning, Christoph Rameshan, Alexander K. Opitz

**Affiliations:** a TU Wien, Institute of Chemical Technologies and Analytics, Getreidemarkt 9/164-EC 1060 Vienna Austria harald.summerer@tuwien.ac.at; b TU Wien, Institute of Materials Chemistry, Getreidemarkt 9/165-PC 1060 Vienna Austria

## Abstract

A new type of catalyst preparation yields its active sites not by infiltration but exsolution of reducible transition metals of its own host lattice. These exsolution catalysts offer a high dispersion of catalytically active particles, slow agglomeration, and the possibility of reactivation after poisoning due to redox cycling. The formation of exsolved particles by partial decomposition of the host lattice can be driven by applying a sufficiently reducing atmosphere, elevated temperatures but also by a cathodic bias voltage (provided the host perovskite is an electrode on an oxide ion conducting electrolyte). In addition, such an electrochemical polarisation can change the oxidation state and thus the catalytic activity of exsolved particles. In this work, we investigate the electrochemical switching between an active and an inactive state of iron particles exsolved from thin film mixed conducting model electrodes, namely La_0.6_Sr_0.4_FeO_3−*δ*_ (LSF) and Nd_0.6_Ca_0.4_FeO_3−*δ*_ (NCF), in humid hydrogen atmospheres. We show that the transition between two activity states exhibits a hysteresis-like behaviour in the electrochemical *I*–*V* characteristics. Ambient pressure XPS measurements proofed that this hysteresis is linked to the oxidation and reduction of iron particles. Furthermore, it is demonstrated that the surface kinetics of the host material itself has only a negligible impact on the particle exsolution, and that the main impact factors are the surrounding atmosphere as well as the applied electrochemical overpotential. In particular, we suggest a ‘kinetic competition’ between gas atmosphere and oxygen chemical potential in the mixed conducting electrode and discuss possible ways of how this process takes place.

## Abstract 2

Unravelling the mystery behind the surface state of exsolved iron nano catalysts for H_2_ oxidation/H_2_O splitting by combining electrochemistry and ambient pressure XPS.

## Graphical abstract

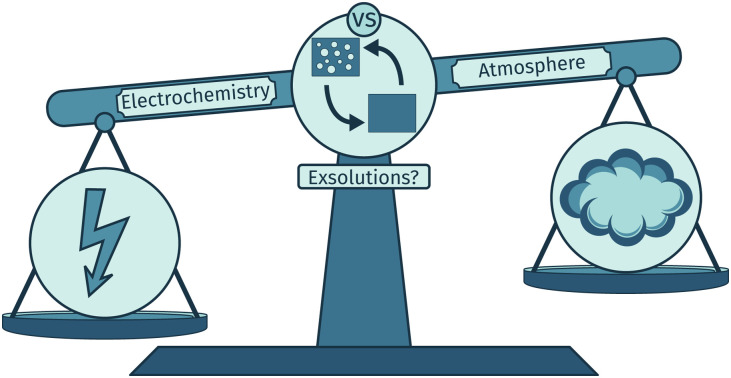

**Broader context**

Heterogeneous catalysis plays a major role in environmentally relevant processes, but the actual preparation of active and stable oxide-supported metal catalysts often proves to be difficult. A novel catalyst preparation method called exsolution is based on creating highly active metallic nanoparticles *via* partial decomposition of a perovskite-type parent oxide. This route allows formation of metal particles with largely decreased agglomeration tendency, thus making them extremely interesting. If the parent perovskite is a mixed conducting electrode in a solid oxide cell, the exsolved particles show another property: upon applying a bias voltage, they can be switched between a metallic and an oxidic state. The associated reversible switchability of catalytic activity can offer unprecedented opportunities for catalysis. Herein, we focus on this electrochemical activity switching and aim at describing the behaviour of the whole system (parent oxide/particles/atmosphere) by the effective chemical potential of oxygen in all three phases. This is not only relevant to unravel the principles behind the switching behaviour, but also provides a basic toolkit for understanding the nature of reactions in non-equilibrium atmospheres (such as water–gas shift) on this novel catalyst type, which can also lead to gradients in the oxygen chemical potential between perovskite, particle, and gas phase.

## 1. Introduction

The increasing utilisation of intermittent renewable energy sources, such as wind or solar, and their entailing variations in power output require reliable storage possibilities.^1–4^ One option is the highly efficient conversion of electrical energy directly into chemical energy *via* electrolysis of water or carbon dioxide in solid electrolysis cells (SOECs),^5–8^ while also allowing the possibility of reverse operation for electricity generation (solid oxide fuel cells, SOFC).^9–11^ The most common cathode (SOEC) or anode (SOFC) materials are Ni/YSZ cermets, which provide both high electronic and ionic conductivity as well as high catalytic activity.^12–14^ However, the metallic particles in these composite electrodes tend to agglomerate after extended time at operation temperature, suffer from sulfur poisoning and can catalyse carbon fibre formation in hydrocarbon atmospheres that may lead to cell damage.^15–19^ Thus, replacement options have been explored to avoid these drawbacks.

Perovskite-type oxides (ABO_3_) and its derivatives have emerged as a popular alternative due to their inherent stability and compositional flexibility. However, their electrochemical performance without any additional activation steps mostly pales in comparison with state-of-the-art fuel electrodes. The most common activation measure is the deposition of electro-catalytically active metal particles *via* infiltration.^20–22^ Due to limited control of the particle and oxide support interaction during and after deposition, these catalysts tend to agglomerate at elevated temperatures or deactivate by coking in the presence of hydrocarbons.^23,24^ A novel way to address these issues is the inclusion of a catalytically active transition metal on the B-site of the perovskite, either as dopant or regular lattice ion. After exposure to sufficiently high temperatures and a strongly reducing atmosphere it is possible to exsolve highly dispersed and catalytically active nanoparticles to the surface of the host perovskite oxide *via* deliberate partial decomposition.^25–37^ Moreover, the obtained particles are socketed on the surface leading to attachment and hence lower agglomeration tendency. This process can also be reversible depending on the applied temperature and atmosphere, consequently allowing repeated reactivation of poisoned metal particles. While this concept has first been explored for exsolution of platinum group metals for automotive exhaust control,^38–40^ there is now ongoing research for a wide range of applications including SOEC cathode materials with various transition metals (*e.g.* Ni,^41–47^ Fe,^42,44,45,47–50^ Co,^44,49,50^ Cu,^51,52^ Mn^44^).

It was shown that the highest nanoparticle density is achieved by fast heating in strongly reducing conditions.^53^ This requirement can partly be avoided by using electrochemical cells that carry the exsolution active perovskite as a working electrode. The application of a bias voltage across the cell changes the oxygen chemical potential of the working electrode and therefore the effective oxygen partial pressure in the working electrode bulk. Thus, a sufficiently strong cathodic overpotential can act as a replacement for highly reducing atmospheres.^54^ Furthermore, the reduction process can be reversed by changing the direction of the applied potential accordingly (*i.e.* more oxidising conditions). As a result, the oxidation state of the exsolved particles and the electro-catalytic activity of the perovskite-type working electrode can be controlled by applying the respective electrochemical polarisation. This concept of “electrochemical activity switching” has already been demonstrated by various authors^51,55–60^ on different electrochemical systems.

Water electrolysis/hydrogen oxidation^55,61^ is a reaction that is technologically highly relevant and its kinetics can be used to determine the catalytic activity of an electrode decorated with exsolutions. In eqn (1) this reaction proceeding on a La_0.6_Sr_0.4_FeO_3−*δ*_ (LSF) electrode is expressed in Kröger–Vink notation:1
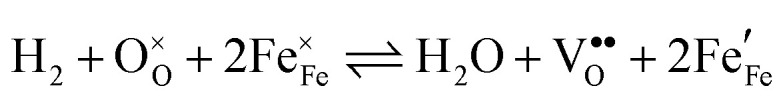
Besides the gaseous species H_2_ and H_2_O, lattice oxide ions (O^×^_O_), regular lattice iron (Fe^×^_Fe_, *i.e.* Fe^3+^), reduced iron (
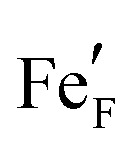
, *i.e.* Fe^2+^) and oxygen vacancies (V^••^_O_) are involved in this reaction. Depending on whether iron exsolutions are present in a reduced and hence metallic or oxidised state, the reaction in 1 proceeds *via* two different mechanisms. If the particles are oxidised it occurs entirely on the perovskite surface with oxidative H_2_ adsorption as the rate determining step. But as soon as the particles are reduced to Fe^0^ they provide a fast bypass for the rate limiting step *via* dissociative H_2_ adsorption on the metal and spillover of the adsorbed hydrogen species to the perovskite surface.^61^ Consequently, if switching the oxidation state of the particles is done by electrochemical polarisation, a step-change in the recorded current–voltage (*I*–*V*) curve can be observed.

In this contribution, we aim to further improve our understanding of the electrochemical switching behaviour of exsolved iron particles on perovskite-type model electrodes and the resulting effects on the H_2_ oxidation/H_2_O splitting kinetics by combining electrochemical impedance spectroscopy (EIS) and *I*–*V* measurements with surface sensitive near ambient pressure – X-ray photoelectron spectroscopy (NAP-XPS). To learn about the impact of the host perovskite's surface kinetics on the switching behaviour of exsolved particles, two different electrode materials – LSF^54,62^ and Nd_0.6_Ca_0.4_FeO_3−*δ*_ (NCF)^63^ – were studied. Moreover, we addressed questions regarding the effects of the oxygen partial pressure in the surrounding atmosphere as well as of the duration and magnitude of an applied cathodic overpotential on the first occurrence of exsolved iron particles. A detailed investigation of the particle's electrochemical switching behaviour over a large atmospheric oxygen partial pressure range allows valuable insights into possible pathways of their electrochemically driven oxidation or reduction. The usage of voltage as a control parameter also permits decoupling of processes, which would inherently be linked in conventional heterogeneous catalysis of non-equilibrium reactions and thus offers an explanation for at first glance unexpected catalyst particle behaviour.

## 2. Experimental methods

### 2.1 Sample preparation

One side polished yttria stabilised zirconia (YSZ) single crystals (Crystec, Germany) with a size of 10 × 10 × 0.5 mm^3^ and (100) orientation were used as electrolyte substrates. Prior to the working electrode, the porous counter electrode consisting of a triple layer was prepared. For this, a thin gadolinia doped ceria (GDC) layer was applied on the unpolished side of the single crystal by spin-coating a slurry consisting of Ce_0.9_Gd_0.1_O_2−*δ*_ powder (*d*_50_ = 250 nm; Treibacher, Austria) and an Terpineol based ink vehicle (www.fuelcellmaterials.com). After drying, a second layer of custom made Platinum-GDC paste was brushed on top of the GDC. A thin Pt layer was applied by brushing Pt paste (Tanaka, Japan) as the last layer. To obtain a 3D porous counter electrode, the dried sample was then sintered in air at 1150 °C for 3 h. This type of counter electrode is known to provide very low ASR in reducing atmospheres.^64^

**Fig. 1:**
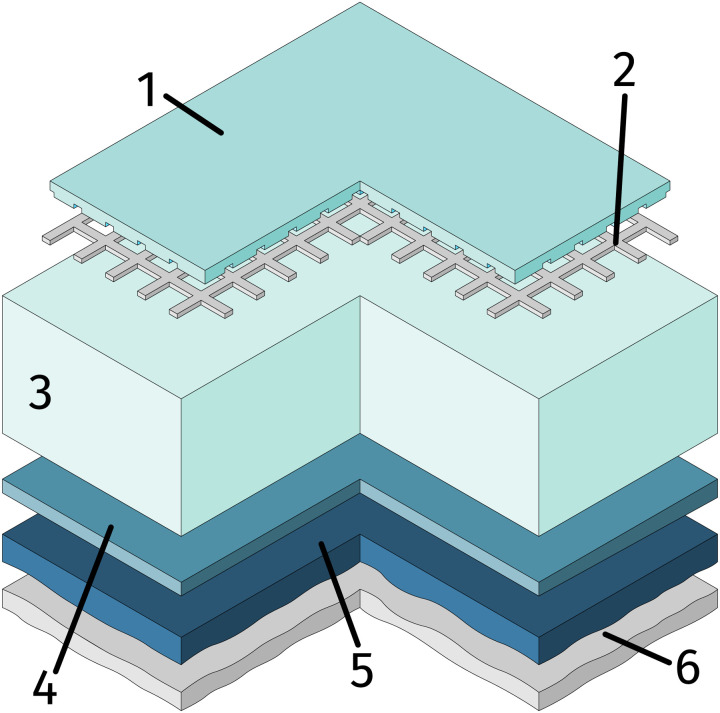
Sample sketch: mixed conductor (thin film) (1), buried Pt current collector (2) grid, yttria stabilised zirconia electrolyte (3), and the counter electrode consisting of GDC (spin-coated) (4), Pt-GDC (brushed) (5), Pt (brushed) (6).

As the first step of the working electrode preparation, a Pt current collector grid (15/5 μm mesh/strip width) was prepared on the polished side of the YSZ crystal by deposition of a 5 nm Ti (adhesion agent) and 100 nm Pt thin film *via* magnetron sputtering (BAL-TEC MED 020) followed by photolithography and ion beam etching. Subsequently, dense thin film working electrodes of either La_0.6_Sr_0.4_FeO_3−*δ*_ (LSF) or Nd_0.6_Ca_0.4_FeO_3−*δ*_ (NCF) were produced by pulsed laser deposition (PLD). The sample was then broken into four pieces of roughly 5 × 5 × 0.5 mm and the sample edges were ground off to avoid parasitic current paths due to deposited Pt or mixed ionic and electronic conducting (MIEC) materials. A sketch of the resulting sample is shown in Fig. 1.

All PLD targets were prepared *via* modified Pechini syntheses.^65^ Appropriate quantities of the precursors La_2_O_3_, SrCO_3_, Fe, CaCO_3_ (all Merck, 99.995%) and Nd_2_O_3_ (Alfa Aesar, 99.995%) for the specific targets were dissolved in nitric acid and citric acid was added in a molar ratio of 1.2 with respect to the total moles of cations. The solution was heated and water was evaporated until self-ignition and combustion of the formed foam took place. The resulting powder was calcined in air at 850 °C for 3 h. Targets for PLD were prepared by uni-axially pressing this powder (150 MPa) and sintering the obtained pellets in air at 1250 °C for 12 h. X-ray diffraction (XRD) of these targets confirmed phase purity. The thin film deposition *via* PLD was achieved by ablating these targets with a KrF excimer laser (Complex Pro 201F, 248 nm) at 600 °C substrate temperature and an O_2_ background pressure of 0.04 mpar with a substrate to target distance of 6 cm. The laser fluence on the target amounted to about 1.3 J cm^−2^, which results in 200 nm films for 18 000 pulses fired at 10 Hz.

### 2.2 *Ex situ* electrochemical impedance spectroscopy (EIS) and *I*–*V* measurements

**Fig. 2:**
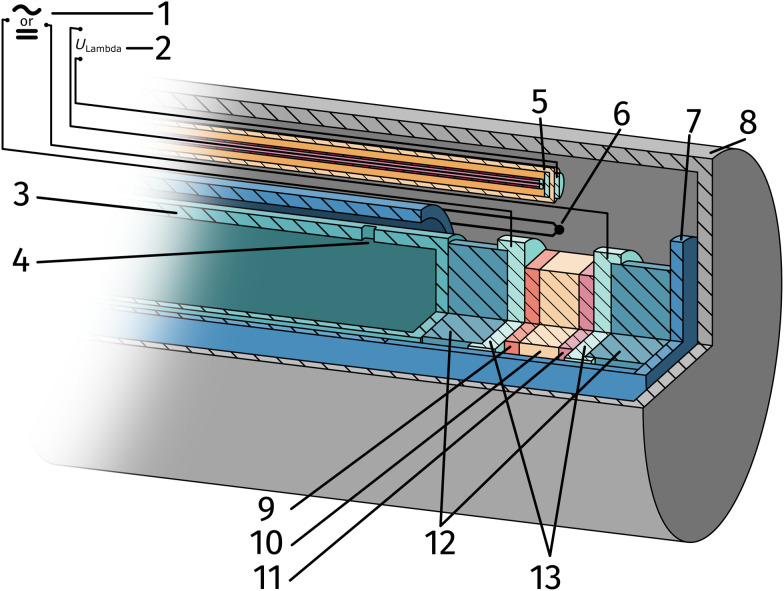
Sketch of the *ex situ* measurement setup: impedance analyser or DC source meter (1), multimeter measuring *U*_Lambda_ (2), inner tube (3), gas inlet (4), custom lambda probe consisting of a hollow YSZ-pipe with Pt-contacts at sample height (5), thermocouple (6), spring loaded middle tube (7), sealed outer tube (8), sample consisting of counter electrode, electrolyte and working electrode (9–11), silica spacer (12) and Pt contact sheets and mesh (13).

The setup employed for the electrochemical *ex situ* measurements is shown in Fig. 2. The sample was clamped between two contacting stamps each consisting of Pt mesh (for simultaneously providing good contact and gas supply), Pt sheet, and a silica spacer utilising a spring loaded middle tube and a fixed inner tube. The latter also served as a gas inlet for various mixtures of H_2_/H_2_O/balance Ar with nominal mixing ratios ranging from H_2_ : H_2_O = 100 : 1 to 1 : 100. A type-S thermocouple was placed close to the sample for temperature measurement. After sealing the setup with a silica outer tube the whole structure was placed in a tube furnace and heated to (600 ± 1) °C sample temperature. A custom made lambda probe consisting of a hollow YSZ pipe with Pt-electrodes at both sides of the pipe was mounted at sample height and thus allowed calculation of the actual oxygen partial pressure 
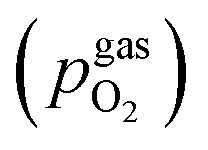
*via* the Nernst equation (*cf.*eqn (2)) with ambient air (inner part of the pipe) as reference.2
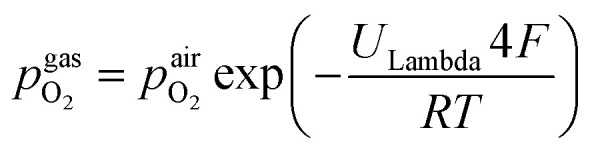
In eqn (2), 
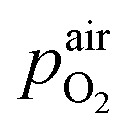
 denotes the oxygen partial pressure in ambient air (209 mpar), *U*_Lambda_ the resulting voltage, *F* Faraday's constant of 96485.34 C mol^−1^, *R* the universal gas constant of 8.314 J K^−1^ mol^−1^ and *T* the operating temperature (873.15 K).

Thin Pt wires connected the aforementioned Pt sheets and the electrical feedthroughs at the cold end of the setup. Electrochemical AC and DC measurements were performed by connecting either a N4L PSM 1735 Frequency Response Analyser with a femto impedance converter or a Keithley 2611A Source Measure Unit to the electrodes. The EIS measurements were carried out before and after each DC measurement block to obtain the YSZ electrolyte resistance and confirm the stability of the working electrode. Spectra were recorded in a frequency range from 1 MHz to 100 MHZ with an AC amplitude of 20 mV.

**Fig. 3:**
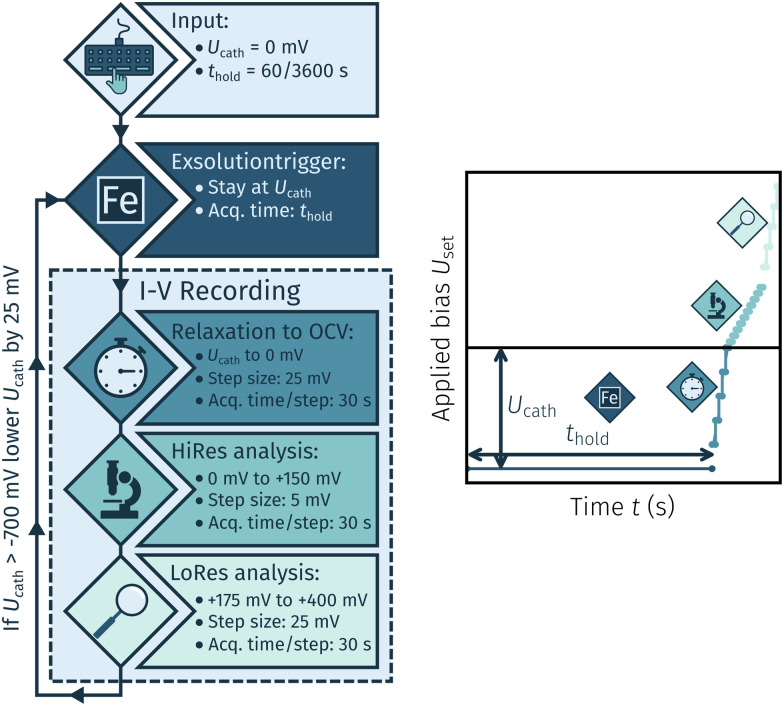
Flowchart of the method used for exsolution onset determination where each measurement cycle consists of three steps: exsolution is triggered by application of a specific cathodic potential *U*_cath_ for a certain acquisition time *t*_hold_. Then an *I*–*V* curve is recorded with varying step sizes. If no step change is observed the applied cathodic potential *U*_cath_ is decreased further for the next iteration.

The *I*–*V* measurements were conducted for two reasons: First, to exsolve the desired catalyst particles, and second, to analyse the electrochemical switching behaviour of these particles under various conditions. The first set of experiments aimed at determining the necessary cathodic potential at which exsolution occurs. The general procedure is shown in Fig. 3. This method encompasses a cycling measurement routine consisting of the following steps:

• A cathodic overpotential *U*_cath_ is applied for a specific holding time *t*_hold_ of either 60 or 3600 s.

• An *I*–*V* curve is recorded by a stepwise *U*-increase +400 mV and 30 s hold time per step. The step size was 25 mV to open circuit, 5 mV between 0 mV and 25 mV again between +175 mV and +400 mV.

• After completion of a full cycle *U*_cath_ is lowered by 25 mV and the whole process is repeated down to a minimum of −700 mV.

As soon as the applied *U*_cath_ triggered the formation of metallic iron exsolutions a dip in the in the anodic branch of the *I*–*V* curve was observed. The overpotential calculated from the respective *U*_cath_ value is denoted as the exsolution onset potential. This routine was repeated with new samples with varied *t*_hold_, electrode materials (LSF and NCF) and H_2_ : H_2_O mixing ratios (10 : 1 and 30 : 1).

The second set of experiments dealt with the electrochemical switching process itself, with a focus laid on the effect of the surrounding atmosphere on the required overpotential to successfully switch the particles between a metallic and an oxidic state. Therefore, *I*–*V* characteristics of samples with already exsolved particles were recorded in different atmospheres and voltage ranges (*U*_set_) between −400 to +400 mV. The step size was either 10 or 20 mV depending on the region of the *I*–*V* curve. Those parts of the *I*–*V* characteristics where the switching point was expected were recorded with a higher resolution. For the rest of the curve a lower resolution was regarded sufficient. The applied atmospheres ranged between H_2_ : H_2_O ≈ 70 : 1 and H_2_ : H_2_O ≈ 1 : 130 with purge times of 2 h between different gas compositions; the exact compositions were measured by the lambda sensor of the setup.

### 2.3 *In situ* NAP-XPS and electrochemical measurements

A detailed description of the setup used for these experiments is beyond the scope of this paper and has already been described in another publication.^66^ In brief, the sample is placed on a ceramic sample holder where it is electrically contacted and held in place by Pt/Ir wires. Inside the measurement chamber of the NAP-XPS system, the water cooled analyser nozzle was moved close (0.5 mm) to the sample and a H_2_ : H_2_O mixture with a ratio of H_2_ : H_2_O ≈ 16 : 1 and a total pressure of 0.75 mbar was introduced. The effective *p*_O_2__ of the atmosphere and thus the H_2_ : H_2_O ratio was calibrated *via* the chemical capacitance of GDC similar to Chueh *et al.*^67^ and also confirmed by mass spectrometry (MS) measurements of the residual gas in the first differential pumping stage of the NAP-XPS system. The sample was heated directly with a near infrared laser through a hole in the backplate of the sample holder.

The X-ray spot size is around the same size as the nozzle diameter of 350 μm. Using an Al-K_α_ source and a monochromator the system achieves a peak width at half maximum of 0.6 eV which corresponds to an energy resolution of 0.2 eV. A Novocontrol Alpha-A High Performance Frequency Analyser (Novocontrol Technologies, Germany) and a Keithley 2611A Source Measure Unit were employed for EIS and *I*–*V* measurements, respectively. The sample temperature was determined from the YSZ electrolyte resistance^68^ in the EIS measurements and set to (600 ± 8) °C. Applying an electrochemical DC polarisation in conjunction with simultaneous XPS measurements allowed us to observe the formation of exsolved particles as well as their switching driven by the applied overpotential virtually in real time.

## 3. Results

### 3.1 Determination of the working electrode overpotential

The overpotential *η*^MIEC^ at the working electrode is calculated by subtracting the bias independent (*i.e.* ohmic) voltage drop at the YSZ electrolyte (given by the product of the resulting current *I*_DC_ and the ohmic resistance *R*_YSZ_) from the applied voltage *U*_set_ as shown in eqn (3).

3 *η*^MIEC^ = *U*_set_ − *I*_DC_*R*_YSZ_

A possible overpotential drop at the counter electrode can be safely neglected due to its extremely low polarisation resistance, as described in ref. 64. The electrolyte resistance *R*_YSZ_ was determined from the measured impedance spectra, as already shown in similar studies, *e.g.* ref. 54 and 69–71 The overpotential obtained *via*eqn (3) directly corresponds to the change in the chemical potential of oxygen *μ*_O_ between the MIEC working electrode and the counter electrode with the latter remaining in equilibrium with the surrounding atmosphere. The resulting working electrode overpotential *η*^MIEC^ for a given atmosphere with a specific 
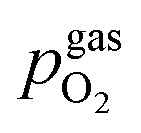
 is thus connected with the difference in the oxygen chemical potential between mixed conducting electrode and gas Δ*μ*^MIEC-gas^_O_ by eqn (4).^54,72^ Here, *F* denotes Faraday's constant and 2 is the number of transferred electrons for eqn (1) being the surface reaction. Consequently, for the present case of surface limited kinetics of the MIEC working electrode, *η*^MIEC^ can be viewed as the thermodynamic driving force of the electrode surface reaction, as already described in other studies.^54,72^

4 Δ*μ*^MIEC-gas^_O_ = *zFη*^MIEC^

The effective 
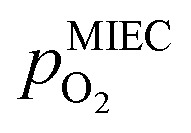
 in the MIEC can therefore be calculated by Nernst's equation given in eqn (5).5
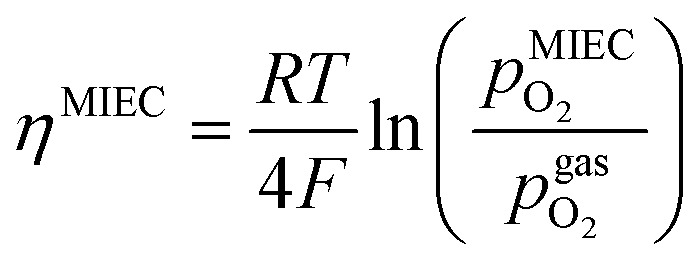


### 3.2 Exsolution onset determination

**Fig. 4:**
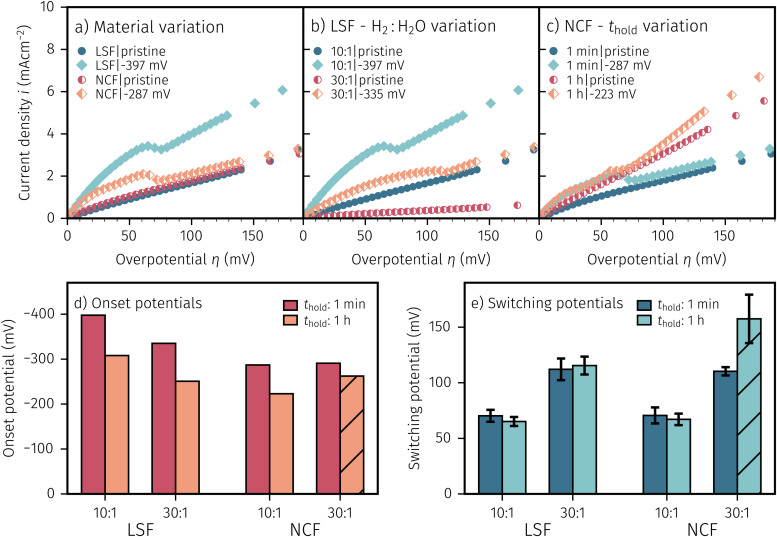
Exsolution onset determination with different (a) materials (LSF, NCF), (b) atmospheres (H_2_ : H_2_O = 10 : 1, 30 : 1) or (c) *t*_hold_ (1 min or 1 h). Each of the figures show the anodic branch of the *I*–*V* characteristics of a pristine sample (without any activity changes) and one after the first visible discontinuity in the anodic current (with the legend caption showing the necessary *U*_cath_). (d and e) Summary of the switching and onset potentials of all varied parameters. The switching potential of a species for a given atmosphere remains unchanged. An increase in *t*_hold_ or a more reducing atmosphere result in a lower onset potential. The hatched sample had different manufacturing conditions (see text for further information).

After applying a sufficiently high cathodic overpotential, a drop in the anodic part of the *I*–*V* curve was observed, as shown in Fig. 4. Previous ambient pressure XPS^55,73^ and XRD^61^ measurements have shown that this drop occurs due to the electrochemically driven oxidation of catalytically active Fe^0^ particles to a less active Fe_1−*x*_O phase. Please note that in the following Fe_1−*x*_O will be abbreviated with FeO for the sake of simplicity. Therefore, the occurrence of this activity drop is a specific and easily measurable characteristic for the existence of a sufficient amount of exsolved Fe^0^ nanoparticles.

In literature, most studies on the exsolution formation conditions focus on temperature and partly on the gas phase, whereas the effect of electrochemical polarisation is – with few exceptions^56^ – largely unexplored. Hence, the question whether the oxides' defect chemistry (which is influenced by the voltage) or the gas phase composition plays the bigger role in the formation of metal exsolutions is not fully resolved.

For better understanding, we studied the impact of the electrode material, the present atmosphere and the duration of the applied cathodic potential on the first occurrence of exsolved particles. Fig. 4a–c show the anodic branches of both a pristine sample (*i.e.* without any cathodic overpotential applied) and after the first appearance of said activity change for exemplary variation of perovskite host material, H_2_ : H_2_O ratio (*i.e.*
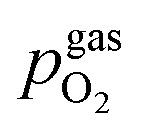
) and holding time of the cathodic voltage to trigger exsolution, respectively. In these graphs, the appearance of an activity switching can be nicely identified. While on pristine samples only regular non-linear *I*–*V* curves could be recorded, electrodes decorated with exsolved particles exhibit a sudden drop of the measured current, despite a further increase of the applied anodic overpotential. This is in line with results from a previous study, where this special behaviour was confirmed to be related to the oxidation of Fe^0^ particles.^61^

The overpotential of activity switching was determined from the activity switching feature as the average between the local maximum before, and the local minimum after said step change. While this may seem arbitrary at first glance, it is important to note that the switching potential obtained *via* this procedure also corresponds with the occurrence of an anomaly (*e.g.* current surges) in the *i vs. t* curves recorded at the respective overpotential (see Subsection 3.4 for further details).

In general, this parameter variation was conducted for all combinations of materials, H_2_ : H_2_O ratios and *t*_hold_. Regardless of the varied parameter, pristine samples (*i.e.* without exsolutions) show lower current densities and LSF seems to be more active compared to NCF. The effect of the performed variations on the exsolution onset potential is summarised in Fig. 4d. From this image it can be concluded that a higher H_2_ : H_2_O ratio and a longer *t*_hold_ lead to a lower onset potential. Interestingly, NCF electrodes also showed formation of exsolved particles already at slightly lower cathodic overpotentials. The switching potential (Fig. 4e), however, appears to be virtually independent of the electrode host oxide materials, which is an interesting observation that will be discussed in a broader context in Section 4. More reducing conditions require higher anodic overpotential to oxidise the exsolved particles, while *t*_hold_ has no impact on the step change of already existing particles. It is worth mentioning at this point that several factors in the sample preparation, such as laser fluence or deposition temperature during the PLD process, can have noteworthy impact on the exsolution onset and switching behaviour (see the hatched bar in Fig. 4d and e). Accidentally, one of the NCF samples was prepared with a different laser fluence leading to slightly deviating results. This may be due to resulting differences in film morphology, stoichiometry, or strain. However, a detailed investigation of the effect of deposition parameters on the exsolution behaviour is beyond the scope of this study and will be the topic of a later publication.

### 3.3 NAP-XPS

**Fig. 5:**
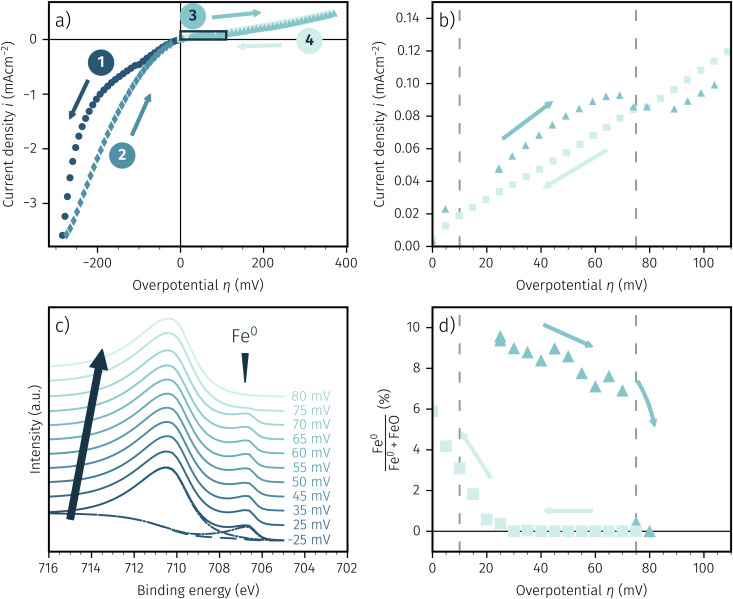
(a) *In situ* recorded *I*–*V* curve for LSF in H_2_ : H_2_O ≈ 16 : 1 with the arrows and numbers indicating measurement direction. (b) Zoom in on the part of the activity change (marked by the rectangle in (a)). (c) Fe 2p XPS spectra consisting of two species: One oxidised species at 710.5 eV and a metallic species at 707 eV (position indicated by the wedge). The large arrow indicates the recording direction. (d) Amount of Fe^0^ compared to the total amount of surface iron based on the applied overpotential.

To *in situ* investigate the change in oxidation state of the exsolved particles when passing through the hysteresis, we conducted NAP-XPS measurements during an electrochemical activity switching experiment. Thus, we could observe the chemical state of the iron surface species based on the applied overpotential as well as recording direction. Owing to technical restrictions of the used NAP-XPS setup the working atmosphere was set to H_2_ : H_2_O ≈ 16 : 1 with at total pressure of 0.75 mbar. Fig. 5a shows the resulting *I*–*V* characteristics for LSF and Fig. 5b depicts a zoom in on the region of the *I*–*V* curve with a hysteresis due to activity switching. In Fig. 5c Fe 2p XPS spectra recorded at increasing overpotentials are shown exemplarily, whereas Fig. 5d shows the percentage of Fe^0^ compared to the total amount of surface iron based on the applied overpotential.

The shape of the electrochemical *I*–*V* curve recorded in the NAP-XPS setup is almost identical with those obtained under *ex situ* conditions in H_2_ rich atmosphere. Still, a strongly asymmetric *I*–*V* characteristic with steep cathodic branch and hysteresis in the anodic region is visible. The only obvious difference is the lower absolute current density, that can be most likely explained by the lower total gas pressure in the XPS chamber.

The Fe 2p spectra were fitted with the software CasaXPS, utilising a shirley background. We chose a simple and robust peak model consisting of two asymmetric Lorentzian peak shapes (LF(0.8, 2.5, 0) and LF(0.6, 3, 10) respectively) for oxidised and metallic iron. Due to the simplistic nature of this model the absolute numbers of the quoted fit results may be subject to somewhat larger uncertainties. However, the fit approach used brings the advantage that a reliable detection of the metallic and oxidic Fe species can be ensured even in the case of relatively strongly changing spectra. The fit is shown in Fig. 5c for the lowest spectra. In this case of an increasing overpotential, there is clearly a metallic iron species visible up to +70 mV, which nicely corresponds to the step change in the *I*–*V* characteristics (see triangles in Fig. 5b). The aforementioned hysteresis is clearly visible in Fig. 5d with the arrows indicating the measurement direction of increasing and subsequent decreasing overpotential with the latter showing almost no surface iron and thus low electrochemical activity. This fits perfectly with the *I*–*V* characteristics of Fig. 5b and is indicated by the dashed lines in both pictures. Hence, we can summarise that the XPS spectra confirm the activity switching hysteresis depending on the measurement direction observed in electrochemical experiments in a previous publication.^61^

### 3.4 Oxygen partial pressure dependence of the electrochemical switching potential

As already briefly shown above, the *p*_O_2__ of the surrounding atmosphere significantly affects the overpotential at which already exsolved surface iron particles can be switched between metallic and oxidic state. Consequently, the oxygen partial pressure dependence was investigated in more detail. This was done in two different ‘regions’, one where Fe^0^ is stable at OCV and the switching potential is expected to appear in the anodic branch of the *I*–*V* curve and one where Fe^0^ is stable only below OCV and said switching potential is located in the cathodic branch.

**Fig. 6:**
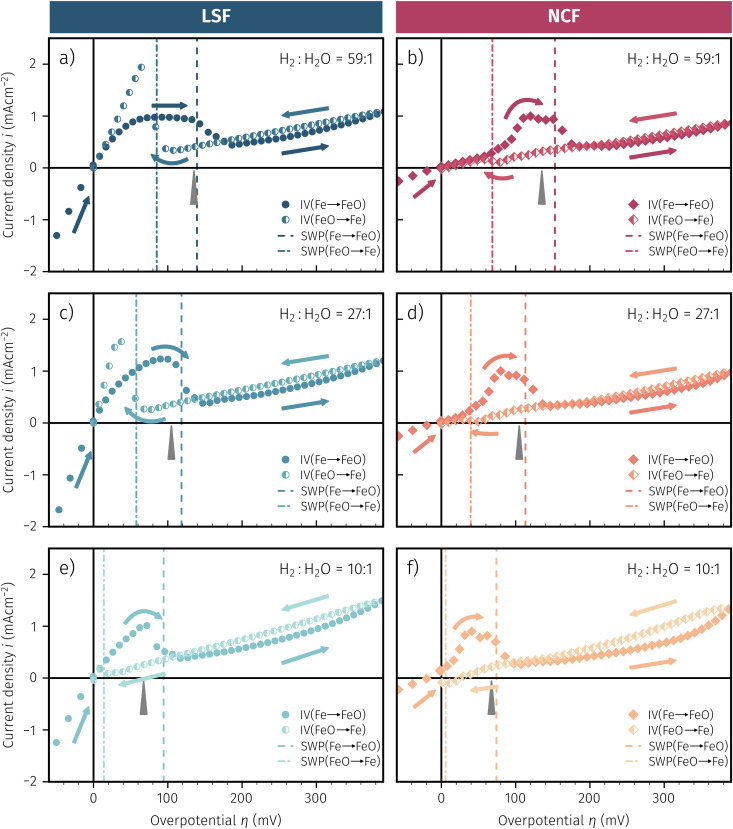
*I*–*V* Characteristics of LSF and NCF at very low oxygen partial pressures (specified as the ratio between H_2_ and H_2_O with increased H_2_ content) showing the current step change due to oxidation or reduction of the exsolved iron particles in both directions (indicated by the dashed/dotdashed line). The arrows specify the measurement direction. The grey wedge represents the thermodynamically expected switching potential (see Section 4 for more information).

Selected examples for an electro-catalytic activity switching behaviour in the anodic branches for both materials (LSF and NCF) at different 
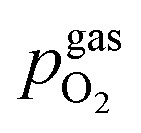
 are shown in Fig. 6. Starting with cathodic bias, the applied voltage is raised up to a maximum of +400 mV (full markers) and then lowered back to open circuit (half-full markers) – the measurement direction is further indicated by arrows. The grey wedge represents the switching potential based on the assumption that the particle is in equilibrium with 
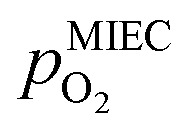
 according to thermodynamic data.^61^ In contrast to an accordingly sharp switching potential a strong hysteresis is visible with a shift of the current step depending on the measurement direction. Thus, two switching potentials can be defined and are shown by the dashed (‘Fe → FeO’-direction) and dotdashed (‘FeO → Fe’-direction) vertical lines in Fig. 6.

As already mentioned in Subsection 3.1, the switching potential shifts to higher anodic values as the oxygen partial pressure decreases, which occurs regardless of the recording direction. It appears that while the exsolved particles are metallic they remain in this state approximately until the thermodynamic transition point, while the re-reduction upon decreasing the applied anodic polarisation lags behind.

**Fig. 7:**
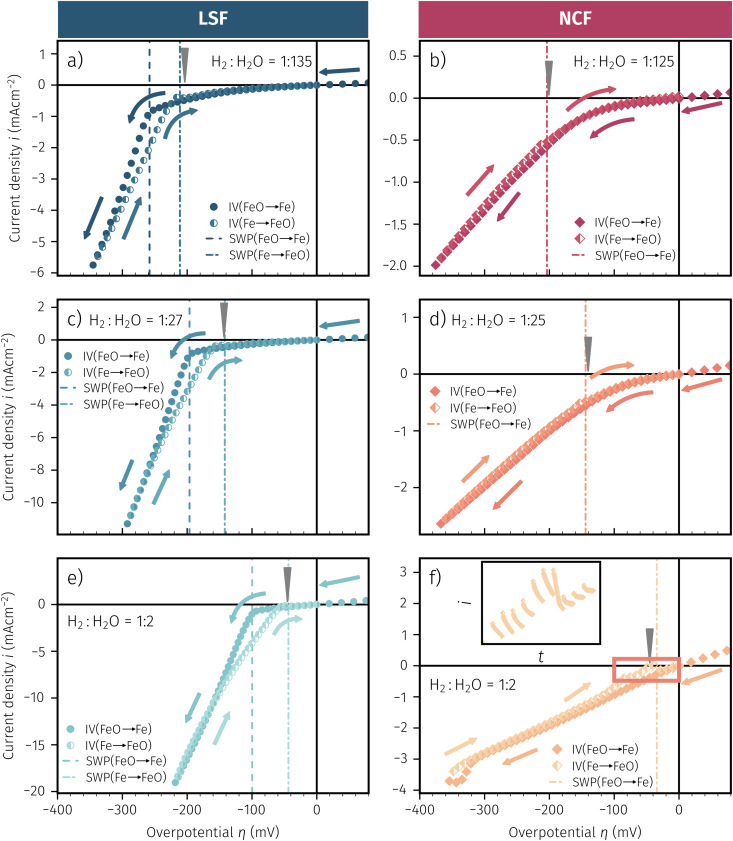
*I*–*V* Characteristics of LSF and NCF at very low oxygen partial pressures (specified as the ratio between H_2_ and H_2_O with increased H_2_O content) showing the current step change – if there is one – in both directions (indicated by the dashed/dotdashed line). The arrows specify the measurement direction. The grey wedge represents thermodynamically expected switching potential (see Section 4 for more information). The inset in (f) represents the current density over time for the highlighted region.

If the switching potential is in the cathodic branch, its position is not as straightforwardly detectable as this is the case for a switching potential in the anodic branch. Selected examples for both materials are depicted in Fig. 7, which is similarly structured like Fig. 6. Colour, marker and arrows indicate recording direction, grey wedge the thermodynamically expected switching potential and the dashed or dotdashed vertical lines the experimentally obtained switching potential for each direction, respectively.

For LSF, the cathodic branch is rather steep indicating high activity with current densities slowly decreasing as the H_2_O content increases. The same applies to NCF but with much lower absolute values. Around the thermodynamic switching potential of Fe/FeO the current drops sharply after reaching a local maximum, which we attribute to the oxidation of the exsolved particles. The catalyst then remains in this low activity state even at large anodic overpotentials. Reversing the measurement direction reveals a shift to lower overpotentials for the re-reduction, visible as a sharp current density increase. In case of determining the switching potential in the ‘Fe → FeO’-direction, *i.e.* strongly cathodic bias back to open circuit, the procedure remains the same as for switching potentials in the anodic branch of the *I*–*V* curve. This only applies fully for LSF. For NCF, especially at higher 
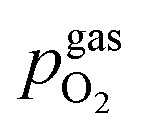
 no clear maximum or minimum is visible. Considering the *i versus t* data (see the inset in Fig. 7f for an example) with the aforementioned current surges, however, allowed determination of said activity change. The reasons for the appearance of this anomaly in the *i vs. t* diagram of the metal to oxide transition of the particles may be associated with the chemical capacitance of the particle.^74,75^ To stay consistent, this second method was compared to the first one for the whole set of data with an apparent local current maximum and resulted in no significant deviations.

For one part of the data, neither current anomalies nor local extreme values were observable, which were the curves in Fig. 7 measured in ‘FeO → Fe’ direction. For LSF, at least a clear bending of the curve indicates an activity change. Thus, for LSF parts of the ‘FeO → Fe’ curve – shallow and steep – were fitted with a linear model separately and the intersection point was defined as the switching potential. While this method was expected to be the least accurate, it actually matches the other two rather well as will be seen in Section 4 below. For NCF, however, this method also did not yield reliably objectifiable switching potentials of the ‘FeO → Fe’ curve in Fig. 7. Hence, this point can unfortunately not be determined for NCF under more oxidising conditions (*i.e.* H_2_ : H_2_O < 1).

**Fig. 8:**
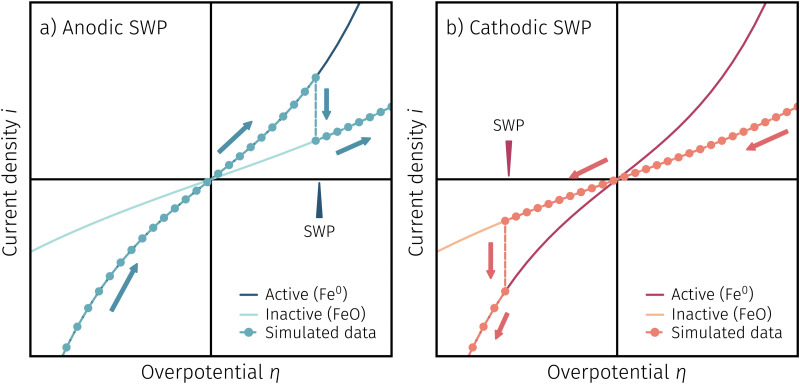
Sketch to explain the difference in switching potential visibility based on measurement direction: (a) an increase in *η* results in a lower *i* which ends up being a real step change. (b) A further decrease in *η* only results in a steeper curve, but no real step change.

One question to be discussed is the disparate visibility of the activity regime change based on measurement direction and/or the location of the switching potential (*i.e.* in the anodic or cathodic branch – compare Fig. 6 and 7, respectively). Two of these scenarios are shown in 8, where the light colours indicate low and dark colours high reaction rates. In Fig. 8a the switching potential is placed in the anodic branch of the *I*–*V* characteristics, which means that the metallic particles oxidise with increasing anodic bias. In this case an actual step change occurs with an apparent ‘peak’ at the position where the current density decreases due to the transition from the high activity to the low activity curve upon increasing anodic overpotential.

In Fig. 8b the switching potential is placed in the cathodic branch and the recording direction chosen to reduce the oxidised particles back to their metallic state. Now, however, a further decrease in the overpotential also yields a decrease in the current density. Thus, the activity regime change does not appear as a peak-like feature, but almost looks like a bend in the resulting *I*–*V* plot.

## 4. Discussion

### 4.1 Possible gradients in the chemical potential of oxygen and their effect on the switching behaviour

In order to explain the occurrence of the hysteresis and its *p*_O_2__ dependence, we have to look at possible reasons for the reduction/oxidation of the surface particles, which take place in this model system. On the one hand, iron oxidation/reduction can happen *via* a chemical reaction with the gas phase (see eqn (6)). This reduction of iron oxide with H_2_ can be modelled with a software like HSC Chemistry, which employs an extensive thermochemical database.^76^ Hence, we are able to obtain the equivalent oxygen partial pressure 
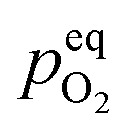
 at which both phases, FeO and Fe, are in equilibrium.

6 FeO + H_2_ ⇌ Fe + H_2_O

7 FeO + 2e^−^ ⇌ Fe + O^2−^

On the other hand, a particle redox reaction is also possible electrochemically by an overpotential at the working electrode (see eqn (7)). In this case the involved electrons and oxide ions are transferred *via* the mixed conducting perovskite oxide. The driving force for eqn (7) is the difference in the chemical potential of oxygen between particle and mixed conductor (Δ*μ*^MIEC-part.^_O_; with *μ*_*O*_ = Δ*μ*_O^2−^_ − 2*μ*_e^−^_). In other words, oxygen is pumped in or out of the particle electrochemically *via* the interface with its parent oxide. The difference between eqn (6) and (7) is essentially the electrochemical H_2_ oxidation/H_2_O splitting reaction (eqn (8)).

8 H_2_ + O^2−^ ⇌ H_2_O + 2e^−^

**Fig. 9:**
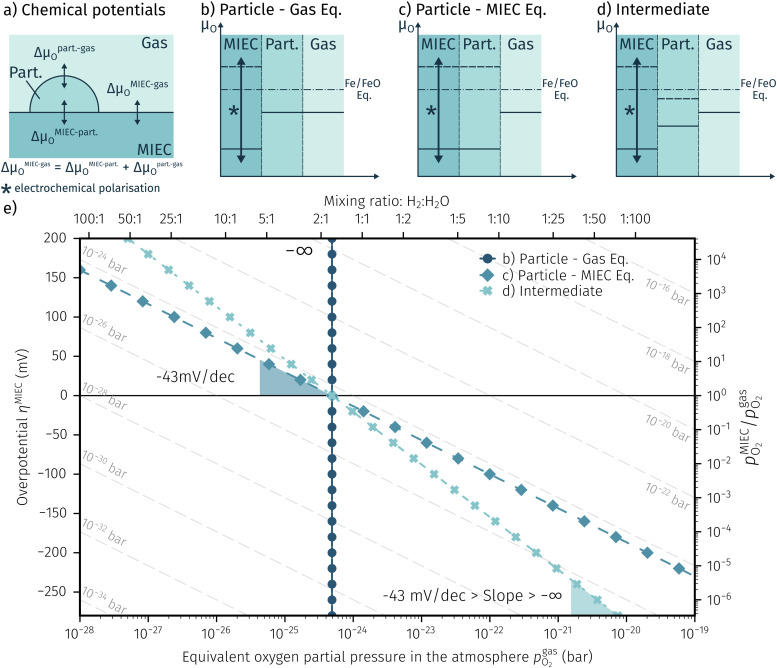
(a) Sketch of all gradients in the oxygen chemical potential Δ*μ*_*O*_ of the involved phases. (b–d) Three different possibilities for the switching behaviour where either Δ*μ*^MIEC-gas^_O_ ≈ Δ*μ*^MIEC-part.^_O_ (b) or Δ*μ*^MIEC-gas^_O_ ≈ Δ*μ*^part.-gas^_O_ (c) applies as well as an intermediate one with both, MIEC and gas, affecting the particle (d). (e) Resulting Fe/FeO equilibria of all three cases shown in a semilogarithmic plot of the overpotential *η* at the working electrode *versus* the equivalent oxygen partial pressure 
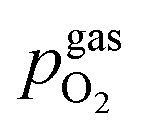
 in the atmosphere. The dashed lines represent the isobars of 
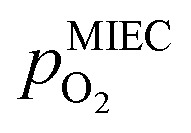
.

As the particle has an interface with both gas and MIEC parent oxide (see Fig. 9a), it is also exposed to both effective oxygen partial pressures, and hence the oxygen chemical potential in the particle is affected by both chemical potentials *μ*^MIEC^_O_ and *μ*^gas^_O_. Depending on whether the kinetics of the chemical reaction eqn (6) or of the electrochemical reaction eqn (7) is faster, the chemical potential of oxygen in the exsolved particles is either mainly governed by the atmosphere or the perovskite-type electrode, respectively. So to speak, the exsolved particles with their *μ*^part.^_O_ are caught between two stools, as sketched in Fig. 9a.

Consequently, it is also possible to formulate a Nernst equation for the particle (eqn (9)), which is not necessarily identical with the one for the perovskite-type MIEC electrode.9
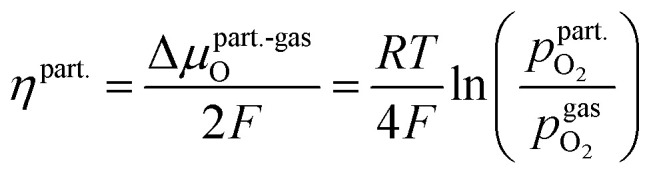
For the experimentally observable switching behaviour at least three different possibilities exist, which depend on the kinetics of both reactions (eqn (6) and (7)) and hence on the established step changes in the *μ*_*O*_ at the particle surface and at its interface with the perovskite electrode. These three cases are depicted in Fig. 9d. In the first case, the oxygen chemical potential of the particle sticks to the one of the gas (*μ*^gas^_O_ ≈ *μ*^part.^_O_) and hence Δ*μ*^MIEC-gas^_O_ ≈ Δ*μ*^MIEC-part.^_O_ holds. In the second case, *μ*^part.^_O_ is only affected by the MIEC (*μ*^MIEC^_O_ ≈ *μ*^part.^_O_; Δ*μ*^MIEC-gas^_O_ ≈ Δ*μ*^part.-gas^_O_) and therefore also by the applied voltage. The third option is an intermediate case, where the particle is influenced by both, the mixed conductor and the surrounding atmosphere.

To visualise the different particle redox behaviour, the expected activity switching potentials (*i.e.* Fe ↔ FeO transition) of the three cases are compared in Fig. 9e. This graph shows the working electrode overpotential *versus* the equivalent oxygen partial pressure in the gas phase 
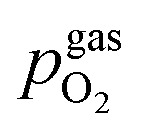
 (on a log scale). As *η*^MIEC^ can also be converted into a 
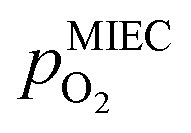
, the ratio between MIEC electrode and gas atmosphere is also given at the right *y*-axis of this plot. The grey dashed lines represent the isobars of 
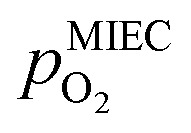
. The expected transition between Fe^0^ metal and Fe-oxide is depicted by the differently coloured lines for all three cases. For each of them, metallic iron is stable on the left hand side of the respective line, while FeO is the stable compound on its right hand side.

### 4.2 Discussion of the experimentally observed catalyst switching

**Fig. 10:**
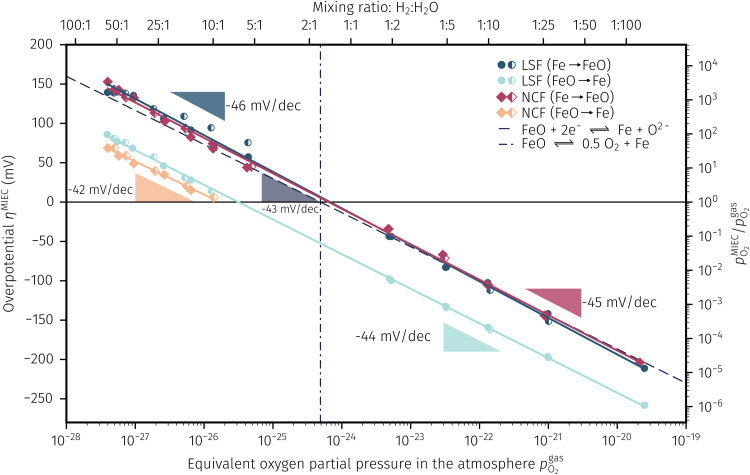
Semilogarithmic presentation of the working electrode overpotential *η versus* the oxygen partial pressure 
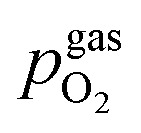
 of the gas analogous to Fig. 9e but including extracted step changes in the *I*–*V* plot for both mixed conducting electrode materials, LSF and NCF. Further distinctions are made with regards to the recording direction of the *I*–*V* curves (see legend) and the 
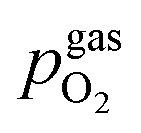
 of the previous *I*–*V* recording, *i.e.* a higher (filled markers) or a lower (half-full markers) one.

In Fig. 10 the same diagram is shown for the experimentally obtained values. In addition, this figure also depicts the Nernst equations for the cases (b) and (c) in Fig. 9, which are given by the thin dotdashed and dashed line (see legend), respectively. The electrochemical activity switching potentials extracted from Fig. 6 and 7 for both materials are separated with respect to the electrochemical recording direction to account for the strong hysteresis. Furthermore, the obtained switching potentials were checked for reproducibility and possible effects due to different prehistory. The filled and half-filled markers indicate that the sample has previously been exposed to lower and higher 
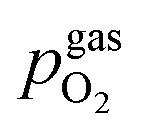
, respectively. Since no significant difference between both data sets (filled and half-filled symbols) can be observed Fig. 10, any effects of long-term degradation or 
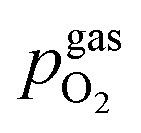
 prehistory can be safely assumed to be negligible.

The results in Fig. 10 allow a number of interesting insights. First, they further illustrate the strong impact of the measurement direction. For switching from the high to the low activity regime (*i.e.* oxidising metallic surface particles to oxides) the switching potentials are significantly higher than the values for the reverse direction, thus reflecting the hysteresis in the *I*–*V* curves - compare ‘Fe → FeO’ with ‘FeO → Fe’ data. Second, no significant difference can be found for LSF and NCF electrodes, thus indicating that the particle redox behaviour is almost independent of the surface kinetics of the parent electrode material. Third, the relative position of the switching potentials reveal valuable details about the kinetics of the particle redox kinetics. For increasing overpotential (‘Fe → FeO’ data) the switching potentials lie almost exactly on the line representing the Nernst equation of the MIEC electrode (*i.e. η*^MIEC^ ≈ *η*^part.^ and hence *μ*^MIEC^_O_ ≈ *μ*^part.^_O_). This means that, when comparing the experimentally found data with the theoretical considerations in Fig. 9e it can be concluded that the second case (Fig. 9c) appears to provide an appropriate model. This would mean that the particles are almost in equilibrium with the parent MIEC, irrespectively of the gas atmosphere - *i.e.* oxygen exchange at the MIEC/particle interface eqn (7) being significantly faster than at the particle/atmosphere interface eqn (6). The slightly higher slope of the ‘Fe → FeO’ curve compared to the theoretical value given by Nernst's equation might be seen as an indication for a small effect of the gas atmosphere and thus the appearance of a small Δ*η* = *η*^MIEC^ − *η*^part.^. Nevertheless, for the switching potentials Δ*μ*^MIEC-gas^_O_ ≈ Δ*μ*^part.-gas^_O_ still holds in very good approximation. Another reason for the observed slight deviation in slope may be associated with a possible socketing effect of the particles on the MIEC electrode. However, owing to the polycrystalline nature of our MIEC,^61^ the data in the current study does not seem to be perfectly suited for making clear statements in this regard, and a quantitative investigation of the influence of the particle-MIEC-interaction on the particle redox behaviour may be the topic of future studies.

In principle, there exists also a further possibility with a dynamic change between the situations in Fig. 9b and c. Let's assume a starting situation of a metallic particle in equilibrium with the gas phase (*i.e.* sketch in Fig. 9b). Initially, the electrochemical pumping rate of oxygen into the particle (*i.e.* net rate of eqn (7) from left to right) is low and thus *μ*^gas^_O_ ≈ *μ*^part.^_O_ holds in good approximation. An increase in the applied anodic overpotential causes an increase of the pumping rate, while the rate of eqn (6) remains constant. At a certain anodic overpotential, the rate of eqn (7) becomes larger than the one of eqn (6). After this point the situation switches from the one sketched in Fig. 9b to the one in Fig. 9c and Δ*μ*^MIEC-gas^_O_ ≈ Δ*μ*^part.-gas^_O_ holds. As long as this transition occurs at a *μ*_O_ below the Fe/FeO equilibrium, the resulting straight in Fig. 9e would be identical to the one of Fig. 9c and thus indistinguishable. Hence, this scenario would also explain the experimental results of ‘Fe → FeO’ measurements in Fig. 10. A discrimination, however, whether the model in Fig. 9c or such a dynamic transition from *μ*^part.^_O_ ≈ *μ*^gas^_O_ to *μ*^part.^_O_ ≈ *μ*^MIEC^_O_ occurs, is not straight forwardly possible from the electrochemical results in this study.

Another rather unexpected peculiarity of the hysteresis is the position of the switching potential from oxidic to metallic Fe-particles. This transition deviates significantly from the thermodynamically expected Fe^0^/FeO equilibrium as can already be seen in Fig. 6 and 7. But even more interesting, by comparing Fig. 9e and 10, it can be deduced that this deviation cannot be explained by any of the three suggested theoretical models. In Fig. 10 this discrepancy appears as the ‘FeO → Fe’ lines running at substantially lower overpotentials as the one predicted by eqn (5). This behaviour is insofar astonishing as it implies that the surface particles remain oxidic even under conditions where both gas phase and MIEC electrode provide a driving force to re-reduce them.

As a reasonable interpretation for the behaviour observed here we suggest a nucleation limitation: A small cluster of Fe^0^ atoms – acting as the nucleus of Fe^0^ re-formation – is less stable than bulk iron, as its formation requires establishing an additional metal/oxide interface, which commonly costs energy. Therefore, a slight overpotential is needed for spontaneous formation and growth of the metallic nucleus. Interestingly, there appears to be little to no overpotential required to nucleate the oxidation reaction. In this case however, no new metal/oxide interface must form, but rather the interface moves into the particle – and thus the surface energy penalty is much lower. What should also be mentioned here is that if this explanation is correct the Fe^0^ particles appear to be sufficiently small that the metal/oxide interface moves through the entire particle quickly enough (based on the findings of a previous paper the metal particle size is a few tens of nm^61^). A systematic investigation of the influence of the particle size on the switching behaviour is currently underway and will be the subject of an upcoming paper.

Another possibility is the formation of Fe_3_O_4_ at high enough overpotentials, as already confirmed in a previous study.^61^ A slower oxide ion diffusion in Fe_3_O_4_ compared to FeO and thus a significant transfer resistance for oxide ions at the Fe_3_O_4_/FeO or Fe_3_O_4_/MIEC interface would in principle also be able to explain the observed discrepancy and is supported by literature data.^77,78^ The particle thus lags behind the effective *p*_O_2__ in the MIEC upon reducing the anodic overpotential past the point of the Fe^0^/FeO equilibrium. This interpretation is also in accordance with the already observed effect, that the occurrence of the hysteresis also has a strong time relation.^61^ Beyond that, also strain in the particles may cause deviations in the particle redox behaviour from the thermodynamically ideal response.

### 4.3 Comprehensive summary of the individual phases of electrochemical activity switching

**Fig. 11:**
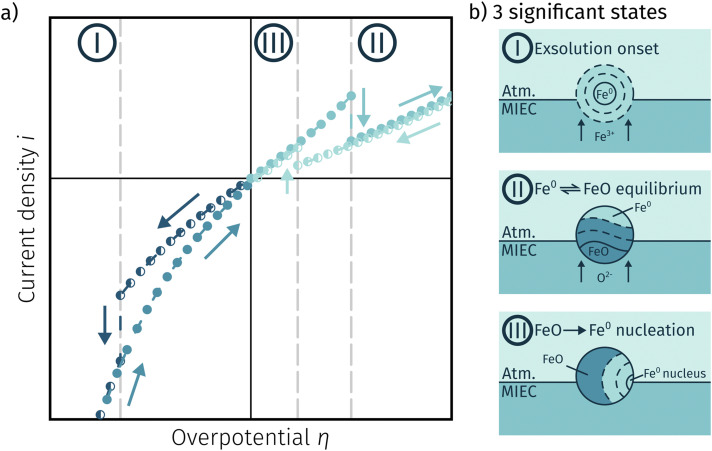
(a) Simulated *I*–*V* curve with a switching potential in the anodic branch showing a large hysteresis defining three significant points (schematic explanation in (b)) based on the measurement direction and the dominant reaction taking place.

Fig. 11a depicts an simulated *I*–*V* curve for a given atmosphere with a switching potential in the anodic branch and a strong hysteresis for the ‘Fe → FeO’ and ‘FeO → Fe’ recording direction. The previous results suggest three significant points. The first one (I in Fig. 11) corresponds to the part of the *I*–*V* curve, where a metallic particles are formed due to a large electrochemical driving force (*i.e.*eqn (7) from left to right).

The regime created by these growing particles is characterised by high electro-catalytic activity and remains present until the electrochemical overpotential pushes the oxygen chemical potential in the particle beyond the thermodynamic threshold for the Fe^0^/FeO transition. As *μ*^MIEC^_O_ ≈ *μ*^part.^_O_ holds in good approximation for the current system, the oxidation of Fe^0^ occurs at the overpotential, where the effective oxygen pressure in the MIEC (described by eqn (5)) is in accordance with the respective *p*_O_2__ of the Fe^0^/FeO equilibrium. Upon oxidation of the exsolved iron particles, the current drops sharply due to less H_ad_ spilling over from the now oxidic particle (point II in Fig. 11).

By again decreasing the applied anodic potential, however, the re-reduction of oxidic particles is shifted to a lower anodic overpotential (point III in Fig. 11) and thus forms the hysteresis. This is also supported by the low current density in the *I*–*V* characteristics as well as the XPS results shown above. We suggest this shift of the point of Fe-oxide particle reduction to originate from a kinetic hindrance due to a nucleation limitation, and the overpotential is needed to facilitate spontaneous formation and growth of the metallic phase. An alternative (but less likely) explanation would be the confirmed formation of Fe_3_O_4_ which exhibits a slower oxide ion diffusion than FeO leading to the particle lagging behind the MIEC after the reduction of the oxygen pumping rate.

### 4.4 Relevance for redox-type catalysts in heterogeneous catalysis

It should be explicitly mentioned that the conclusions drawn in the previous sections are not only relevant for exsolution-decorated oxide electrodes but also for conventional heterogeneous catalysis on these novel catalysts. The main difference between conventional heterogeneous and electrochemically supported catalysis is that the usage of voltage as a controllable parameter allows decoupling of processes which are tied together otherwise. For example, in a non-equilibrium gas atmosphere - like it is the case for a mixture of H_2_ and CO_2_ that can react *via* the well-known reverse water–gas shift reaction (rWGS; H_2_ + CO_2_ → H_2_O + CO) – the effective oxygen partial pressure is ill-defined. Whether such a non-equilibrium gas mixture has a reducing or an oxidising effect on a material, depends on the respective reaction kinetics. On an oxide supported metal catalyst, this may lead to the situation that the effective oxygen partial pressure in the particle differs from the one in the MIEC parent oxide. When studying the activity of an exsolution catalyst for such a reaction, it may not be easily recognisable that the actual chemical state of the particles may be mainly controlled by the kinetics of oxygen exchange between the mixed conducting support and the gas atmosphere (see Fig. 9a for reference). Applying a voltage that causes an electrochemical stoichiometry polarisation of the MIEC parent oxide offers a convenient possibility to adjust *μ*^MIEC^_O_ and *μ*^gas^_O_ independently from each other, which in this study was the decisive factor for unravelling the complex kinetic interplay between atmosphere, particle, and perovskite-type parent oxide.

## 5. Conclusion

In this work, perovskite-type ferrite materials were used as model-systems to successfully gain insights into the formation of catalytically active Fe^0^ exsolutions as well as the electrochemical switching behaviour of these catalyst particles. Dense LSF and NCF thin films on YSZ electrolytes with fast GDC based counter electrodes were prepared and their exsolution behaviour was investigated with special emphasis on the chemical/oxidation state of the obtained surface particles and their impact on the H_2_ oxidation/H_2_O electrolysis reaction. This was achieved by a combination of electrochemical (mainly *I*–*V* characteristics) and surface sensitive methods (*in situ* NAP-XPS). The measurements revealed the following:

• The recorded *I*–*V* curves showed three distinct points, where sudden step changes in the current density were observable. At sufficiently cathodic overpotentials this is linked to the formation of exsolved Fe^0^ nanoparticles, which can then be oxidised in the anodic branch after reaching the Fe^0^/FeO equilibrium. The re-reduction, however, is hampered by the Fe^0^ nucleation process resulting in a hysteresis.

• For the initial formation of exsolution particles, lower effective *p*_O_2__ values, stronger cathodic polarisation and longer holding times were found to act promoting. The composition of the parent oxide and its prehistory-dependent surface state apparently played a significant role for the kinetics of H_2_ oxidation/H_2_O splitting, however, the latter shows only a minor (if any) correlation with particle exsolution.

• Once exsolved, the particles could be reversibly oxidised and reduced by changes of the applied overpotential and atmosphere. Aided by theoretical considerations, we could show that not the atmospheric composition but the oxygen chemical potential in the perovskite bulk is decisive for the redox state of the particles.

• For re-reduction of the particles, interestingly none of the theoretical models reflects the observed behaviour, but rather a phase transition at more reducing conditions as the thermodynamically expected overpotential was found, which may be due to a nucleation limitation related to the re-formation of a metal/oxide interface.

• The independent adjustment of *μ*^MIEC^_O_ and *μ*^part.^_O_ with the applied voltage allowed decoupling of the relevant processes, which control the surface state of exsolved particles, thus providing an important contribution to the understanding of heterogeneous catalysis on exsolution catalysts.

## Conflicts of interest

There are no conflicts to declare.
